# Changes of Cytokines in Saliva of Pigs with *Streptococcus suis* Infection Measured with a Multiplex Assay

**DOI:** 10.3390/vetsci12040316

**Published:** 2025-03-31

**Authors:** Alberto Muñoz-Prieto, Luis Pardo-Marín, Elena Goyena, Edgar García Manzanilla, José Joaquín Cerón, Lorena Franco-Martínez

**Affiliations:** 1 Interdisciplinary Laboratory of Clinical Pathology, Interlab-UMU, Department of Animal Medicine and Surgery, Campus of Excellence Mare Nostrum, University of Murcia, 30100 Murcia, Spain; alberto.munoz@um.es (A.M.-P.); lpm1@um.es (L.P.-M.); jjceron@um.es (J.J.C.); 2 Department of Anatomy and Comparative Anatomy, Campus of Excellence Mare Nostrum, University of Murcia, 30100 Murcia, Spain; goyena@um.es; 3 Moorepark Animal and Grassland Research Center, Teagasc, Irish Agriculture and Food Development Authority, Cork P61 C996, Ireland; edgar.garciamanzanilla@teagasc.ie

**Keywords:** biomarkers, cytokines, multiplex, sialochemistry, porcine, oral fluid, saliva, *Streptococcus suis*

## Abstract

**Experimental Objective:** This study evaluated potential changes in cytokines in the saliva of pigs with meningitis and *Streptococcus suis* (*S. suis*) infection, using a multiplex assay. **Methods:** For this purpose, levels of 13 cytokines, namely GM-CSF, IFNγ, IL-1α, IL-1β, IL-1ra, IL-2, IL-4, IL-6, IL-8, IL-10, IL-12, IL-18, and TNF-α, were measured in porcine saliva from 29 male weaning piglets (6 to 9 weeks old) with meningitis and *S. suis* infection (*S. suis* group, n = 15) and healthy controls (healthy group, n = 14). Samples were measured using Luminex xMAP^®^ technology with a multiplex assay that was analytically evaluated in this study. **Results:** The measurement of these cytokines provided adequate values during the analytical validation conducted in this study, except for GM-CSF, TNF-α, IFNγ, and IL-8. Particularly for GM-CSF and TNF-α, many values, especially in healthy pigs, fell below the limit of quantification of the assay. In pigs with *S. suis* infection, 11 out of the 13 cytokines, namely GM-CSF, IFNγ, IL-1α, IL-1β, IL-2, IL-4, IL-6, IL-10, IL-12, IL-18, and TNF-α, showed statistically (*p* < 0.05) higher levels compared to the healthy controls. **Conclusions:** These findings underscore an association between *S. suis* infection and alterations in selected cytokine levels in the saliva of pigs afflicted with this disease, thus highlighting the potential role of these cytokines in the immune response to infection.

## Simple Summary

This study evaluates for the first time the measurement and comparison of cytokine levels in the saliva of healthy pigs and pigs with meningitis positive to *Streptococcus suis* (*S. suis*), using a multiplex assay. A total of 13 cytokines—GM-CSF, IFNγ, IL-1α, IL-1β, IL-1ra, IL-2, IL-4, IL-6, IL-8, IL-10, IL-12, IL-18, and TNF-α—were quantified, and the analytical performance of the assay was assessed. The validation showed acceptable results, except for GM-CSF, TNF-α, IFNγ, and IL-8. Specifically, GM-CSF and TNF-α levels, particularly in healthy pigs, were often too low to be accurately measured. In pigs infected with *S. suis*, 11 out of the 13 cytokines (GM-CSF, IFNγ, IL-1α, IL-1β, IL-2, IL-4, IL-6, IL-10, IL-12, IL-18, and TNF-α) were higher than in healthy controls (n = 15 vs. n = 14, respectively). These findings suggest that porcine saliva could serve as a valuable fluid for detecting cytokines involved in the immune response to infection and for distinguishing between healthy and infected animals.

## 1. Introduction

*Streptococcus suis* (*S. suis*) infection is a challenging problem in pig breeding, having a zoonotic potential [1,2]. It is caused by a Gram-positive bacterium and is associated with significant mortality and morbidity in pigs, resulting in an average mortality of 4–12% among weaning piglets [3,4,5], causing major economic losses in the swine industry [6]. *S. suis* is present in several locations shortly after birth, including the saliva [7], palatine and nasopharyngeal tonsils, and mandibular lymph nodes [4,8] in healthy animals [9,10]. However, *S. suis* can cause disease with a variety of clinical manifestations, including arthritis, pneumonia, endocarditis, or meningitis, which is the most common manifestation [5,11]. Most cytokines are produced very early in infectious diseases and were proposed as helpful for evaluating their severity [12,13,14]. In the case of *S. suis*, some components of its bacterial cell wall induce the production of pro-inflammatory cytokines, including TNF-α, interleukin (IL)-1β, IL-6, and IL-8, in murine macrophages [15] and human THP-1 monocytes [16]. In vitro studies in porcine blood have revealed the induction of pro-inflammatory cytokines by *S. suis*, specifically TNF-α, IL-1β, and IL-6 [17], while a previous report described increases in blood IL-6 and IL-10 levels after an experimental intravenous infection with *S. suis* serotype 2 [18]. However, information remains limited on in vivo data concerning the relationship between induced cytokines and *S. suis* in pigs and, to the best of the author’s knowledge, cytokines have not been evaluated in saliva for this disease.

Saliva sampling contrasts with other analytical samples, such as blood or cerebrospinal fluid, which require invasive and, especially in the case of cerebrospinal fluid, technically complex procedures. Saliva sampling in pigs and other farm animals is usually an easy, non-invasive procedure that minimizes stress, making it suitable for large-scale studies, as it can be safely collected by non-specialized personnel, including the animals’ caretakers [19]. Thus, the use of saliva facilitates research in large-animal models in general and porcine models in particular [20]. In the case of *S. suis* infection, a previous proteomic study revealed alterations in saliva between pigs with meningitis caused by *S. suis* and healthy controls [21]. These alterations suggest disruptions in cytokine-associated pathways, including the innate immune system, hemostasis, neutrophil degranulation, and platelet degranulation, signaling, and aggregation. In this line, increases in salivary biomarkers linked to immune response and inflammation in pigs with *S. suis* infection, in addition to changes in biomarkers associated with stress, redox status, and muscle damage, have also been reported [22]. These studies highlight the potential of saliva for evaluating inflammation and immune response in this disease.

To the best of our knowledge, there are no studies exploring the possible cytokine changes in the saliva of pigs with meningitis caused by *S. suis*. Thus, the main objective of this study was to investigate a panel of 13 cytokines in saliva in response to *S. suis* infection in piglets. For this purpose, a commercially available immunoassay kit designed to measure cytokines in pigs was validated for its application in saliva and utilized in this study.

## 2. Material and Methods

### 2.1. Animals and Sample Collection

The present study evaluated surplus saliva samples from a previous one [21]. In brief, samples were obtained from 29 male weaning piglets [*Sus scrofa domesticus* (Large White)], aged 6 to 9 weeks and weighing between 11.3 and 21.7 kg, from a commercial farm located in the region of Murcia, Spain. Animals were divided into two groups: (1) clinically healthy pigs (Healthy, n = 14), which served as the control group, and (2) pigs with naturally occurring meningitis tested positive for *S. suis* (*S. suis*, n = 15). Animals from the *S. suis* group presented clinical symptomatology (ataxia, anorexia, lateral recumbency, and paddling) [23] consistent with meningitis [24] and were positive for *S. suis* serotype 9 as measured by blood PCR [25,26].

Saliva collection from *S. suis* was performed when the clinical symptoms were most evident by using a sponge (45 mm × 25 mm × 25 mm; Esponja Marina, La Griega E. Koronis, Madrid, Spain) clipped to a flexible thin metal rod approximately 20 cm in length, which was exposed to the pigs. After the pigs had thoroughly moistened the sponge by chewing, the sponges were placed into Salivette^®^ tubes (Sarstedt, Aktiengesellschaft and Co., Nümbrecht, Germany) and kept refrigerated until arrival at the laboratory. The Salivette tubes were then centrifuged at 3000× *g* and 4 °C for 10 min, and saliva supernatants were transferred into Eppendorf tubes and stored at −80 °C until analysis.

All procedures were approved by the Ethical Committee on Animal Experimentation (CEEA) of the University of Murcia (protocol code CEEA 563/2019).

### 2.2. Cytokine Validation and Measurements in Porcine Saliva

Cytokine measurement was performed with Luminex xMAP^®^ technology and a multiplex assay, specifically utilizing the MILLIPLEX^®^ MAP kit Porcine Cytokine/Chemokine Magnetic Bead Panel (Cat#PCYTMG-23K-13PX for pig reactivity, Merck Millipore, Burlington, MA, USA), following the manufacturer’s instructions. This kit enabled the measurement of 13 cytokines/chemokines, including granulocyte macrophage colony-stimulating factor (GM-CSF), interferon-gamma (IFNγ), interleukins (IL)-1α, IL-1β, IL-1ra, IL-2, IL-4, IL-6, IL-8, IL-10, IL-12, IL-18, and tumor necrosis factor-α (TNF-α).

First, an array of salivary samples from healthy pigs and pigs with *S. suis* infection were measured. This served to determine if values within the assay’s limits of detection were obtained, to define possible dilution of samples, and to determine which samples were high or low for each cytokine.

Then, samples having high and low values for each cytokine were selected for analytical validation in porcine saliva, based on sample volume availability. The analytical validation of the multiplex assay on porcine salivary samples was assessed through precision and accuracy according to previously described protocols [27,28,29,30]. Coefficients of variation (CV) were assessed to determine the assay’s precision, evaluating intra-assay precision. For this, 3 samples with high and low cytokine concentrations were measured 3 times in the same run (intra-assay determination). Accuracy was evaluated indirectly by performing serial dilutions of 2 high samples using ultrapure water (Millipore) as diluent (1:2, 1:4, 1:8, and 1:16) and analyzed. Spiking recovery was assessed by standard addition methodology [31] to evaluate accuracy. For this, the highest-level assay calibrator standard was added at 50% volume to 1 low sample. The assay calibrator was added without dilution, and then diluted at ratios of 1:2, 1:4, and 1:8. Recovery (in percent) was calculated for each dilution by comparing expected and observed values.

### 2.3. Statistical Analysis

Results are expressed as medians and interquartile ranges. Data was evaluated for normality of distribution using the D’Agostino and Pearson omnibus normality test. As not all the data followed a normal distribution, the Mann–Whitney test was performed to determine possible differences of statistical relevance between healthy pigs and pigs with meningitis. The correlation matrix among all the cytokines was calculated using the non-parametric Spearman correlation test.

Principal component analysis (PCA) was performed using the prcomp() function in RStudio (Version: 2024.04.2+764), calculating correlation matrix, eigenvectors, and eigenvalues. Variable loadings for each principal component were computed to identify key cytokines contributing to group differentiation.

In all cases, differences were considered significant when the *p*-value was below 0.05. Statistics analyses were performed using GraphPad Prism 8 software (Version 8.0.2) (GraphPad Software Inc., La Jolla, CA, USA) and RStudio (RStudio Team, 2022).

## 3. Results

For porcine saliva, all measurements were above the manufacturer’s reported minimum detectable concentration, except for six measures (five from the healthy group) for GM-CSF and two measures (one from the healthy group) for IFNγ determinations. Overall, of the 377 measurements made in this study, only eight (2.1% of the total data, 75% of them from healthy animals’ samples) were below the limits of detection provided by the manufacturer.

Validation results of the assay using porcine saliva are shown in Table 1. The reference material of the kit demonstrated intra-assay CV consistently below 1.7% and spiking recovery rates ranging from 96% to 110.8%. The linearity of these measurements was confirmed through linear regression analysis of the standards, with correlation coefficients (R2) exceeding 0.999 in all instances.

During the analytical validation of saliva using samples with high and low concentrations for each cytokine, all measurements surpassed the minimum detectable concentrations, except for three low samples of GM-CSF and one sample of TNF-α. In these cases, for the statistical study, the values were substituted with the manufacturer’s reported lower limit of quantification (LLOQ).

Linearity under dilution resulted in linear regression equations with correlation coefficients > 0.9 in both analyzed samples for IL-1a, IL-1B, IL-1ra, IL-4, IL-6, IL-8, IL-10, IL-12, and IL-18. However, IFNγ provided low coefficients of correlation (R^2^ < 0.4) in both samples. In the case of GM-CSF, IL-2, and TNF-α, linearity under dilution could not be determined as the values after the first dilution were lower than the limit of quantification.

Intra-assay coefficients of variation for samples with high values were below 15% in all cases, except for one of the three samples used for GM-CSF (24.7%). For samples with low values, intra-assay CVs were less than 15% in most cases, except for two samples of the three used for IL-4 (24.7% and 21.7% CV, respectively). For samples with low values, in one sample for TNF-α and all three for GM-CSF, most measurements were below LLOQ and thus, CV could not be determined.

Finally, spiking recovery using a sample and the highest standard in different dilutions yielded results within ±20% variation for all concentrations in IL-1α, IL-4, and IL-10. In the cases of IL-2 and TNF-α, spiking recovery fell within the 80–120% reference range when the standards were diluted at 1:2 and 1:4 ratios, whereas spiking recovery for IL-1β, IL-1ra, IL-6, IL-12, and IL-18 fell within the 80–120% reference range only when the standard was diluted at 1:4. Finally, in the cases of GM-CSF, IFNγ, and IL-8, spiking recovery was <80%.

Cytokine levels in the saliva of healthy pigs and pigs with meningitis and *S. suis* infection are shown in Table 2 and Supplementary Figure S1. There were statistically significant higher values in *S. suis*-infected individuals with meningitis compared to healthy controls for all analytes, except IL-8 and IL-1ra. For all biomarkers except IL-12, fold changes were >2, with the cytokines showing the highest increases being IL-1α, IL-1ra, and IFNγ with 4.5-, 4.7-, and 6.8-fold changes, respectively.

A correlation analysis was conducted among the various analytes in the samples, revealing a total of 40 statistically significant correlations, accounting for approximately 51% of the total comparisons (Table 3). Among the cytokines, the strongest correlations were observed between IL-1β and IL-8 (r = 0.915, *p* < 0.001), IL-2 and IL-4 (r = 0.876, *p* < 0.001), and IL-6 and IL-18 (r = 0.876, *p* < 0.001).

**Figure 1:**
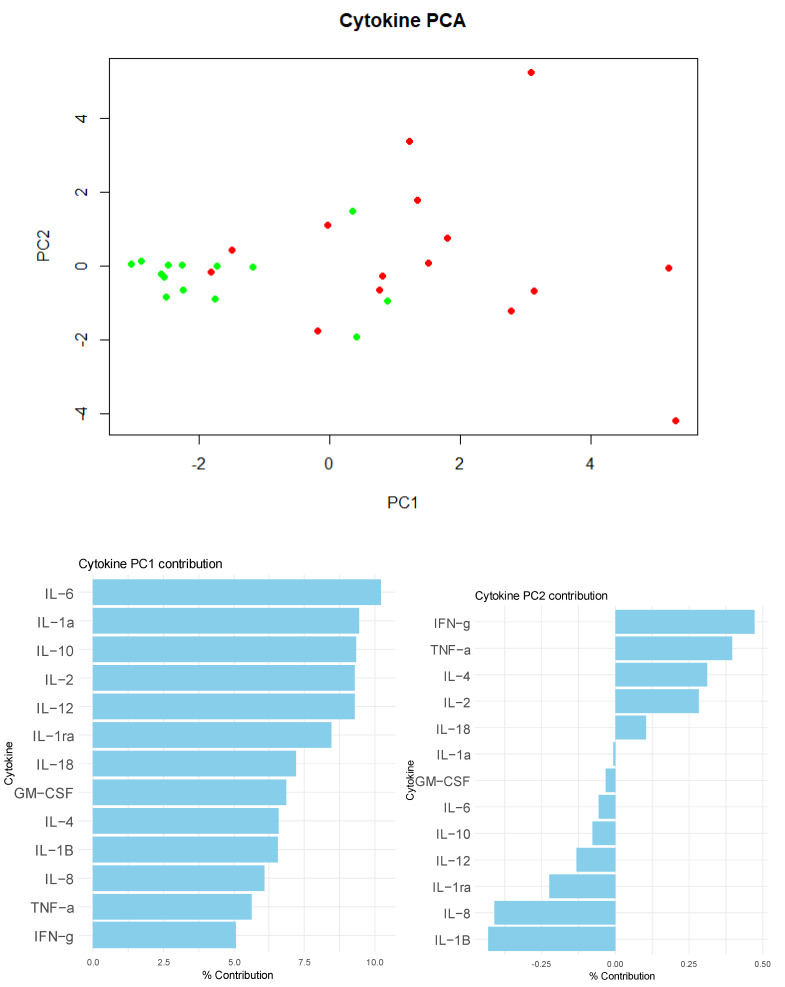
Results of the PCA carried out to unravel specific response patterns among the cytokines evaluated and associate them with *S. suis* infection (results from the healthy group are represented in green, and results from the *S. suis* group are represented in red). The graphs on the left column represent cytokine contributions to principal components 1 and 2, respectively. Cytokines: granulocyte macrophage colony-stimulating factor (GM-CSF †), interferon-gamma (IFNγ †), interleukin (IL)-1α, IL-1β, IL-1ra, IL-2, IL-4, IL-6, IL-8 †, IL-10, IL-12, IL-18, and tumor necrosis factor-alpha (TNF-α †). † GM-CSF, TNF-α, IFNγ, and IL-8 did not yield satisfactory results in the analytical validation.

The PCA (Figure 1) revealed a separation between the two groups, primarily along the first principal component (PC1) axis, which accounted for 43.73% of the total variance. This suggests distinct cytokine profiles between the healthy and meningitis groups. Examining the loadings of the cytokines on PC1, cytokines such as IL-6, IL-1α, and IL-10 contributed significantly to the observed separation, as they exhibited strong loadings in the direction of the PC1 axis. Additionally, the cumulative proportion of variance explained by the first two principal components reached 64.58%, indicating that a substantial portion of the dataset’s variability was captured within these two dimensions.

## 4. Discussion

Cytokines are integral components of the innate immune response, playing diverse roles in inflammation and immune response [32]. They contribute to the overall development and regulation of the inflammatory process, including cell activation and recruitment, antigen presentation, and cell differentiation in the bone marrow [33], making them a valuable source for evaluating infections. To the best of our knowledge, this study is the first to evaluate the modulation of cytokines in porcine saliva in animals with *S. suis* infection.

For cytokine measurements, a Luminex bead array assay was used, enabling simultaneous measurement of several cytokines. This assay has demonstrated acceptable intra-assay reproducibility in porcine serum and seminal plasma [34,35,36]. It was approved by the National Institute of Biological Standards and Control, and the measurements were conducted following the manufacturer’s instructions. As this kit has not been previously employed in porcine saliva, an analytical validation was performed using this specimen. First, saliva samples from healthy and *S. suis*-infected pigs were measured to determine if the kit provided results above the manufacturer’s limit of detection and to identify samples with high and low values for each cytokine for further validation. After confirming that all measurements, except for two samples for IFN-γ and six for GM-CSF, were above the minimum detectable concentrations, the analytical validation was performed. This validation included intra-assay repeatability, linearity under dilution, and spiking recovery using reference material. The intra-assay CV reported acceptable precision except for GM-CSF and TNF-α, for which the results were influenced by the low values observed in saliva samples, with many cases falling below the LLOQ. For this reason, linearity could not be evaluated in these two analytes. The other cytokines, except for IFNγ (showing an R^2^ of 0.38), exhibited adequate linearity after serial sample dilution, with coefficients of determinations R^2^ > 0.9. When the highest standard was added at different concentrations to saliva samples, spiking recovery results were within the ±20% acceptance range for three cytokines (IL-1α, IL-4, and IL-10). For the remaining cytokines, except for GM-CSF, IFNγ, and IL-8, spiking recoveries were within the acceptance range when the standard was diluted (1:2 or 1:4), indicating a potential matrix effect of saliva in these cytokines. This effect could be minimized when samples are diluted to at least 1:2, as recommended by the assay’s instructions. Based on the presented results, the analytical validation indicated that most measurements were within acceptable ranges, particularly for linearity and repeatability. However, IFNγ should be interpreted with caution, as it was not linear and was not adequately detected with the added standard to the saliva samples. Additionally, GM-CSF and TNF-α were below the limit of detection in some samples, especially in healthy animals, which could be attributed to the assay’s lack of sensitivity or to their low concentrations in porcine saliva. Furthermore, IL-8 did not yield acceptable results in the spiking recovery test. As we used saliva from pigs infected with *S. suis* for the analytical validation, one possible explanation for the results obtained with IL-8 could be its cleavage by a serine protease produced by *S. suis* [37].

Our results revealed that the median concentrations of cytokines measured in saliva were at least 1.5-fold higher in pigs with meningitis and *S. suis* infection compared to healthy controls for all 13 cytokines measured, with these differences being of statistical relevance for all analytes except IL-1ra and IL-8. These findings align with previous reports indicating increased cytokine levels in the blood of pigs experimentally infected with *S. suis* serotype 2 (IL-6 and IL-10) [18], as well as with *Neisseria meningitidis* (all five measured cytokines: IL-1β, IL-6, IL-8, IL-10, and TNF-α) [38]. Cytokines are considered biomarkers of severity and could help determine the cause of infection. While plasma IL-6 levels rise more prominently in bacterial infections, IL-8 and IL-18 levels may be even more specific, discriminating between Gram-negative and Gram-positive infections, with IL-8 being higher when the causal agent is a Gram-negative bacterium and IL-18 being higher in Gram-positive infections [39,40,41]. This is consistent with our results in saliva, where animals infected with *S. suis* (Gram-positive bacteria) exhibited statistically higher values than healthy controls in IL-6 (*p* < 0.001) and IL-18 (*p* = 0.001). In contrast, IL-8, which increases more in Gram-negative bacteria, did not show significant changes between groups (*p* = 0.31).

The panel of cytokines analyzed in this study belongs to different families, based on their biological functions and the signaling pathways involved. Of the 13 measured, 11 (GM-CSF, IFNγ, IL-1α, IL-1Β, IL-2, IL-4, IL-6, IL-10, IL-12, IL-18, and TNF-α) exhibited higher concentrations in the saliva of pigs with meningitis and *S. suis* infection. In brief, based on a previous classification [32], four groups of cytokines could be identified: (1) proinflammatory cytokines, such as IL-1α, IL-1β, IL-6, and TNF-α, which are involved in promoting inflammation; (2) anti-inflammatory cytokines, such as IL-1ra and IL-10, dampen inflammatory responses; (3) cytokines that regulate immune responses, such as IL-2, IL-4, IL-12, IL-18, and IFNγ, which play crucial roles in modulating the immune system; and (4) cytokines involved in growth and cell differentiation, such as GM-CSF. Notably, among other correlations, we observed strong (r > 0.65) correlations among cytokines with similar functions. For example, IL-1α correlated with IL-6 and TNF-α (r = 0.671, *p* < 0.001 in both cases), while the anti-inflammatory IL-1ra correlated with IL-12 (r = 0.659, *p* < 0.001). Strong correlations were also observed among immune system modulators, such as between IL-2 and IL-4 (r = 0.876, *p* < 0.001) and IL-18 (r = 0.842, *p* < 0.001), and between IL-4 and IL-18 (r = 0.743, *p*< 0.001). Thus, based on our results, it can be indicated that the higher values of these cytokines in the saliva of infected pigs reflect changes in a variety of functions related to inflammation and the immune system, and the correlations observed between the different cytokines highlight the complex interplay among them.

To further explore the underlying structure of cytokine data and their ability to differentiate between healthy individuals and those diagnosed with meningitis, a PCA analysis was conducted. The PCA analysis revealed that IL-6, IL-1α, and IL-10 were the cytokines that explained most of the total variance and contributed the most to group differentiation, explaining 64.58% of the variance. Therefore, although these two principal components can capture a significant portion of the variability in cytokine profiles between the studied groups, there is still a considerable proportion of unexplained variance. It is expected that, due to the high inter-individual variations, increasing the sample size could improve the PCA’s ability to separate the groups more clearly.

The presence of inflammation during *S. suis* infection has been previously documented, with increases in salivary levels of biomarkers of inflammation, including acute phase proteins (APPs) such as haptoglobin or inter-alpha-trypsin inhibitor heavy chain 4 (ITIH4, Pig-Map) [21,22]. Since cytokines are known to play a crucial role in the early stages of inflammation, being the precursors of APPs, we hypothesized that cytokine levels in saliva may serve as an earlier marker of inflammatory response. The higher levels observed for most analysed cytokines found in saliva in pigs with *S. suis* in comparison to healthy controls suggest that these cytokines could be useful as non-invasive early biomarkers of inflammation during *S. suis* infection. The increase in cytokines could reflect the activation of the immune system and the onset of an inflammatory response, which may later be confirmed by increases in APPs. From a practical standpoint, the use of saliva as a sample for cytokine measurement offers a valuable tool for monitoring inflammation in animals without the need for invasive procedures and using a more convenient sampling method. Therefore, this approach could improve the early detection and monitoring of *S. suis* infection, contributing to better management and diagnosis in field settings.

The present study has some limitations. One limitation is the lack of an experimental infection model to evaluate cytokines in saliva. Such an approach would have allowed the tracking of cytokine dynamics in relation to disease progression in blood, cerebrospinal fluid, and saliva, enabling a direct comparison of cytokine dynamics across these biofluids, while minimizing exposure to other infectious agents. It would also have helped determine when high cytokine levels in saliva are produced (e.g., during colonization or the transition from colonization to invasive disease), providing further insights. However, the primary aim of this study was to assess the feasibility of measuring cytokines in porcine saliva with the assay evaluated and to have a preliminary overview of how these cytokines might change in the case of pigs with an inflammatory condition such as *S. suis* infection, rather than conducting an in-depth investigation of the immune response to *S. suis* infection. Therefore, we opted to use surplus saliva samples from a previous study involving naturally infected piglets from commercial farms, adhering to the 3R principle (Replacement, Reduction, and Refinement) while also evaluating a real-world scenario. Another limitation was that, in our study, blood PCR revealed that all animals with meningitis tested positive for *S. suis* serotype 9, one of the most prevalent serotypes causing clinical disease in Europe [1]. However, future studies analyzing different *S. suis* serotypes, coinfections, or physiological variables such as sex and age are needed to further clarify cytokine responses in saliva and compare them with results obtained from other biofluids. Additionally, it would be of great interest to perform further studies to evaluate the possible use of these cytokines as biomarkers for treatment monitoring and prognosis of the disease. Finally, it is important to point out that the results obtained for the cytokines that did not give satisfactory results at the validation (GM-CSF, IFNγ, IL-8, and TNF-α,) should be interpreted with caution. The feasibility of alternative techniques for the measurement of these cytokines, such as ELISA, or more sensitive assays such as AlphaLISA, for their measurement in saliva should be further explored.

## 5. Conclusions

When a panel of cytokines (including GM-CSF, IFNγ, IL-1α, IL-1β, IL-1ra, IL-2, IL-4, IL-6, IL-8, IL-10, IL-12, IL-18, and TNF-α) was measured in saliva from healthy pigs and in pigs with meningitis and *S. suis* infection, upregulation of several cytokines was observed in the diseased animals. This pattern suggests an inflammatory and immune response elicited by the body against the pathogen. These findings underscore the dynamic interplay between the host immune system and *S. suis*, emphasizing the importance of cytokine regulation in the pathogenesis of *S. suis* infection and opening potential uses and applications for cytokine measurements in saliva for this disease. The use of saliva offers several advantages, including painlessness, reduced stress for both personnel and animals and ease of collection, making it a viable alternative to blood or tissue analysis. However, these results should be interpreted with caution, since some cytokines, such as GM-CSF, TNF-α, IFNγ, and IL-8, did not yield satisfactory results in the analytical validation.

## Figures and Tables

**Table 1:** Analytical validation of cytokines in porcine saliva.

		GM-CSF	IFNγ	IL-1α	IL-1β	IL-1ra	IL-2	IL-4	IL-6	IL-8	IL-10	IL-12	IL-18	TNF-α
**Linearity (R2)**	High A	**LLOQ**	**0.382**	0.988	0.998	0.989	**LLOQ**	0.974	0.982	1.000	0.917	0.968	0.980	**LLOQ**
	High B	**LLOQ**	**0.376**	1.000	0.998	0.901	**LLOQ**	0.949	0.914	1.000	0.980	0.904	0.967	**LLOQ**
**Intra-assay CV (%)**	High A	0.0	13.2	4.6	6.2	2.6	0.0	10.2	3.9	2.9	14.7	0.0	8.0	0.0
	High B	0.0	7.7	3.5	7.1	3.3	0.0	8.7	14.9	2.7	13.9	0.0	5.9	0.0
	High C	**24.7**	14.6	3.9	4.3	2.1	0.0	8.1	3.7	7.5	8.7	0.0	2.7	0.0
	Low A	**LLOQ**	12.6	4.7	0.0	4.2	0.0	**24.7**	0.0	2.2	0.0	0.0	0.0	**LLOQ**
	Low B	**LLOQ**	13.6	5.6	0.0	10.1	0.0	**21.7**	0.0	5.6	0.0	0.0	0.0	0.0
	Low C	**LLOQ**	3.8	1.8	0.0	1.7	0.0	0.0	10.8	0.3	0.0	0.0	0.0	0.0
**Spiking recovery (%)**	Saliva	100.0	100.0	100.0	100.0	100.0	100.0	100.0	100.0	100.0	100.0	100.0	100.0	100.0
	50 + 50	**19.5**	**50.9**	81.1	**50.8**	**68.7**	**75.2**	110.7	**67.4**	**8.9**	82.8	**60.7**	**76.8**	**69.0**
	50 + 50 (1:2)	**37.1**	**26.2**	113.9	**65.7**	**79.4**	92.6	82.9	**71.1**	**13.5**	91.6	**74.9**	**73.0**	95.2
	50 + 50 (1:4)	**61.0**	**59.7**	128.2	80.5	88.3	109.0	106.1	80.5	**25.9**	93.5	89.3	80.1	98.7
	STD	100.0	100.0	100.0	100.0	100.0	100.0	100.0	100.0	100.0	100.0	100.0	100.0	100.0

Linearity coefficient of correlation (R^2^) of two samples with high values. Intra-assay coefficient of variation (%), based on three measurements each of three high-value and three low-value samples. Spiking recovery (%) of one sample with low values with different concentrations of the highest calibration standard. Cytokines: granulocyte macrophage colony-stimulating factor (GM-CSF), interferon-gamma (IFNγ), interleukin (IL)-1α, IL-1β, IL-1ra, IL-2, IL-4, IL-6, IL-8, IL-10, IL-12, IL-18, and tumor necrosis factor-alpha (TNF-α). In bold, R2 < 0.7, CV > 15%, and spiking recovery outside 120–80%. LLOQ: lower limit of quantification. Data in bold highlight parameters outside acceptance ranges.

**Table 2:** Cytokine concentrations in porcine saliva in healthy pigs (Healthy) and pigs with *S. suis* infection (*S. suis*).

Cytokine (ng/mL)	Median (25th, 75th Percentiles)	
	Healthy	*S. suis*
GM.CSF	0.02 (0.02. 0.03)	0.04 (0.02. 0.06) *
IFNγ	0.17 (0.1125. 0.5175)	1.16 (0.26. 2.42) *
IL-1α	0.66 (0.525. 1.6575)	2.96 (1.32. 3.99) ***
IL-1β	0.16 (0.07. 0.55)	0.39 (0.07. 0.64) ***
IL-1ra	5.83 (1.91. 15.0575)	27.61 (1.57. 61.58)
IL-2	0.025 (0.02. 0.0325)	0.06 (0.04. 0.11) ***
IL-4	0.08 (0.0675. 0.12)	0.32 (0.16. 0.53) ***
IL-6	0.14 (0.11. 0.335)	0.42 (0.28. 0.7) ***
IL-8	1.95 (1.0725. 3.6775)	4.48 (0.79. 7.74)
IL-10	0.04 (0.04. 0.06)	0.12 (0.04. 0.19) ***
IL-12	0.02 (0.0175. 0.02)	0.03 (0.02. 0.06) ***
IL-18	0.425 (0.3175. 0.62)	0.94 (0.56. 1.38) **
TNF-α	0.02 (0.02. 0.02)	0.04 (0.03. 0.08) **

Cytokines: granulocyte macrophage colony-stimulating factor (GM-CSF †), interferon-gamma (IFNγ †), interleukin (IL)-1α, IL-1β, IL-1ra, IL-2, IL-4, IL-6, IL-8 †, IL-10, IL-12, IL-18, and tumor necrosis factor-alpha (TNF-α †). Asterisks highlight statistical significance between the groups (*: *p* < 0.05; **: *p* < 0.01; ***: *p* < 0.001). † GM-CSF, TNF-α, IFNγ, and IL-8 did not yield satisfactory results in the analytical validation.

**Table 3:** Cytokine correlation matrix (r; *p*) for porcine saliva, depicted as a heatmap. The color value of the cells is proportional to the strength of the associations, ranging from red (negative correlations) to green (positive correlations).

	GM-CSF	IFNγ	IL-1α	IL-1β	IL-1ra	IL-2	IL-4	IL-6	IL-8	IL-10	IL-12	IL-18	TNF-α
GM.CSF		**r = 0.44 ** ***p* = 0.017**	**r = 0.412 ** ***p* = 0.027**	r = −0.151 *p* = 0.433	r = 0.027 *p* = 0.889	**r = 0.633 ** ***p* < 0.001**	**r = 0.444 ** ***p* = 0.016**	**r = 0.507 ** ***p* = 0.005**	r = −0.092 *p* = 0.634	**r = 0.386 ** ***p* = 0.038**	r = 0.315 *p* = 0.096	r = 0.364 *p* = 0.053	r = 0.326 *p* = 0.084
IFNγ	**r = 0.44 ** ***p* = 0.017**		**r = 0.535 ** ***p* = 0.003**	r = −0.254 *p* = 0.184	r = −0.041 *p* = 0.832	**r = 0.6 ** ***p* = 0.001**	**r = 0.676 ** ***p* < 0.001**	**r = 0.397 ** ***p* = 0.033**	r = −0.264 *p* = 0.166	**r = 0.502 ** ***p* = 0.005**	r = 0.366 *p* = 0.051	**r = 0.438 ** ***p* = 0.018**	**r = 0.614 ** ***p* < 0.001**
IL-1α	**r = 0.412 ** ***p* = 0.027**	**r = 0.535 ** ***p* = 0.003**		r = 0.354 *p* = 0.06	**r = 0.467 ** ***p* = 0.011**	**r = 0.646 ** ***p* < 0.001**	**r = 0.705 ** ***p* < 0.001**	**r = 0.671 ** ***p* < 0.001**	**r = 0.371 ** ***p* = 0.047**	**r = 0.735 ** ***p* < 0.001**	**r = 0.84 ** ***p* < 0.001**	**r = 0.632 ** ***p* < 0.001**	**r = 0.671 ** ***p* < 0.001**
IL-1β	r = −0.151 *p* = 0.433	r = −0.254 *p* = 0.184	r = 0.354 *p* = 0.06		**r = 0.722 ** ***p* < 0.001**	r = 0.051 *p* = 0.793	r = −0.024 *p* = 0.901	r = 0.338 *p* = 0.073	**r = 0.915 ** ***p* < 0.001**	**r = 0.44 ** ***p* = 0.017**	**r = 0.585 ** ***p* = 0.001**	r = 0.243 *p* = 0.204	r = 0.32 *p* = 0.09
IL.1ra	r = 0.027 *p* = 0.889	r = −0.041 *p* = 0.832	**r = 0.467 ** ***p* = 0.011**	**r = 0.722 ** ***p* < 0.001**		**r = 0.38 ** ***p* = 0.042**	r = 0.349 *p* = 0.064	**r = 0.505 ** ***p* = 0.005**	**r = 0.63 ** ***p* < 0.001**	**r = 0.659 ** ***p* < 0.001**	**r = 0.602 ** ***p* = 0.001**	**r = 0.529 ** ***p* = 0.003**	r = 0.359 *p* = 0.056
IL-2	**r = 0.633 ** ***p* < 0.001**	**r = 0.6 ** ***p* = 0.001**	**r = 0.646 ** ***p* < 0.001**	r = 0.051 *p* = 0.793	**r = 0.38 ** ***p* = 0.042**		**r = 0.876 ** ***p* < 0.001**	**r = 0.806 ** ***p* < 0.001**	r = 0.04 *p* = 0.836	**r = 0.653 ** ***p* < 0.001**	**r = 0.612 ** ***p* < 0.001**	**r = 0.842 ** ***p* < 0.001**	**r = 0.631 ** ***p* < 0.001**
IL-4	**r = 0.444 ** ***p* = 0.016**	**r = 0.676 ** ***p* < 0.001**	**r = 0.705 ** ***p* < 0.001**	r = −0.024 *p* = 0.901	r = 0.349 *p* = 0.064	**r = 0.876 ** ***p* < 0.001**		**r = 0.694 ** ***p* < 0.001**	r = −0.039 *p* = 0.84	**r = 0.688 ** ***p* < 0.001**	**r = 0.604 ** ***p* = 0.001**	**r = 0.743 ** ***p* < 0.001**	**r = 0.673 ** ***p* < 0.001**
IL-6	**r = 0.507 ** ***p* = 0.005**	**r = 0.397 ** ***p* = 0.033**	**r = 0.671 ** ***p* < 0.001**	r = 0.338 *p* = 0.073	**r = 0.505 ** ***p* = 0.005**	**r = 0.806 ** ***p* < 0.001**	**r = 0.694 ** ***p* < 0.001**		r = 0.289 *p* = 0.128	**r = 0.638 ** ***p* < 0.001**	**r = 0.633 ** ***p* < 0.001**	**r = 0.87 ** ***p* < 0.001**	**r = 0.591 ** ***p* = 0.001**
IL-8	r = −0.092 *p* = 0.634	r = −0.264 *p* = 0.166	**r = 0.371 ** ***p* = 0.047**	**r = 0.915 ** ***p* < 0.001**	**r = 0.63 ** ***p* < 0.001**	r = 0.04 *p* = 0.836	r = −0.039 *p* = 0.84	r = 0.289 *p* = 0.128		**r = 0.407 ** ***p* = 0.028**	**r = 0.647 ** ***p* < 0.001**	r = 0.197 *p* = 0.305	**r = 0.393 ** ***p* = 0.035**
IL-10	**r = 0.386 ** ***p* = 0.038**	**r = 0.502 ** ***p* = 0.005**	**r = 0.735 ** ***p* < 0.001**	**r = 0.44 ** ***p* = 0.017**	**r = 0.659 ** ***p* < 0.001**	**r = 0.653 ** ***p* < 0.001**	**r = 0.688 ** ***p* < 0.001**	**r = 0.638 ** ***p* < 0.001**	**r = 0.407 ** ***p* = 0.028**		**r = 0.79 ** ***p* < 0.001**	**r = 0.557 ** ***p* = 0.002**	**r = 0.75 ** ***p* < 0.001**
IL-12	r = 0.315 *p* = 0.096	r = 0.366 *p* = 0.051	**r = 0.84 ** ***p* < 0.001**	**r = 0.585 ** ***p* = 0.001**	**r = 0.602 ** ***p* = 0.001**	**r = 0.612 ** ***p* < 0.001**	**r = 0.604 ** ***p* = 0.001**	**r = 0.633 ** ***p* < 0.001**	**r = 0.647 ** ***p* < 0.001**	**r = 0.79 ** ***p* < 0.001**		**r = 0.596 ** ***p* = 0.001**	**r = 0.774 ** ***p* < 0.001**
IL-18	r = 0.364 *p* = 0.053	**r = 0.438 ** ***p* = 0.018**	**r = 0.632 ** ***p* < 0.001**	r = 0.243 *p* = 0.204	**r = 0.529 ** ***p* = 0.003**	**r = 0.842 ** ***p* < 0.001**	**r = 0.743 ** ***p* < 0.001**	**r = 0.87 ** ***p* < 0.001**	r = 0.197 *p* = 0.305	**r = 0.557 ** ***p* = 0.002**	**r = 0.596 ** ***p* = 0.001**		**r = 0.581 ** ***p* = 0.001**
TNF-α	r = 0.326 *p* = 0.084	**r = 0.614 ** ***p* < 0.001**	**r = 0.671 ** ***p* < 0.001**	r = 0.32 *p* = 0.09	r = 0.359 *p* = 0.056	**r = 0.631 ** ***p* < 0.001**	**r = 0.673 ** ***p* < 0.001**	**r = 0.591 ** ***p* = 0.001**	**r = 0.393 ** ***p* = 0.035**	**r = 0.75 ** ***p* < 0.001**	**r = 0.774 ** ***p* < 0.001**	**r = 0.581 ** ***p* = 0.001**	

Cytokines: granulocyte macrophage colony-stimulating factor (GM-CSF †), interferon-gamma (IFNγ †), interleukin (IL)-1α, IL-1β, IL-1ra, IL-2, IL-4, IL-6, IL-8 †, IL-10, IL-12, IL-18, and tumor necrosis factor-alpha (TNF-α †). Data in bold highlight statistical significance (*p* < 0.05). † GM-CSF, TNF-α, IFNγ, and IL-8 did not yield satisfactory results in the analytical validation.

## Data Availability

The datasets used in this study are available from the corresponding author upon reasonable request.

## Supplementary Materials

The following supporting information can be downloaded at: https://www.mdpi.com/article/10.3390/vetsci12040316/s1, Figure S1: Cytokine determinations (ng/mL) in porcine saliva in the group of animals with S. suis infection compared with healthy controls. Individual values are represented as dots and horizontal lines indicated median and 25th-75th percentiles values. Cytokines: Granulocyte macrophage colony-stimulating factor (GM-CSF †), interferon-gamma (IFNγ †), interleukin (IL)-1α, IL-1β, IL-1ra, IL-2, IL-4, IL-6, IL-8 †, IL-10, IL-12, IL-18, and tumor necrosis factor-alpha (TNFα †). Asterisks highlight statistical significance between the groups (*: *p* < 0.05; **: *p* < 0.01; ***: *p* < 0.001). † GM-CSF, TNF-α, IFNγ and IL-8 did not yield satisfactory results in the analytical validation.
