# Supplementary material for: Changes of Cytokines in Saliva of Pigs with Streptococcus suis Infection Measured with a Multiplex Assay
Source: Vet Sci. 2025 Mar 31;12(4):316. doi: 10.3390/vetsci12040316 (PMC12031078; doi:10.3390/vetsci12040316)
Supplement: Supplementary file 1 [file vetsci-12-00316-s001.zip › vetsci-3496112-supplementary.pdf]

# Supplementary materials: Changes of Cytokines in Saliva of Pigs with *Streptococcus suis* Infection Measured with a Multiplex Assay

**Alberto Muñoz-Prieto, Luis Pardo-Marín, Elena Goyena, Edgar García Manzanilla, José Joaquín Cerón and Lorena Franco-Martínez**

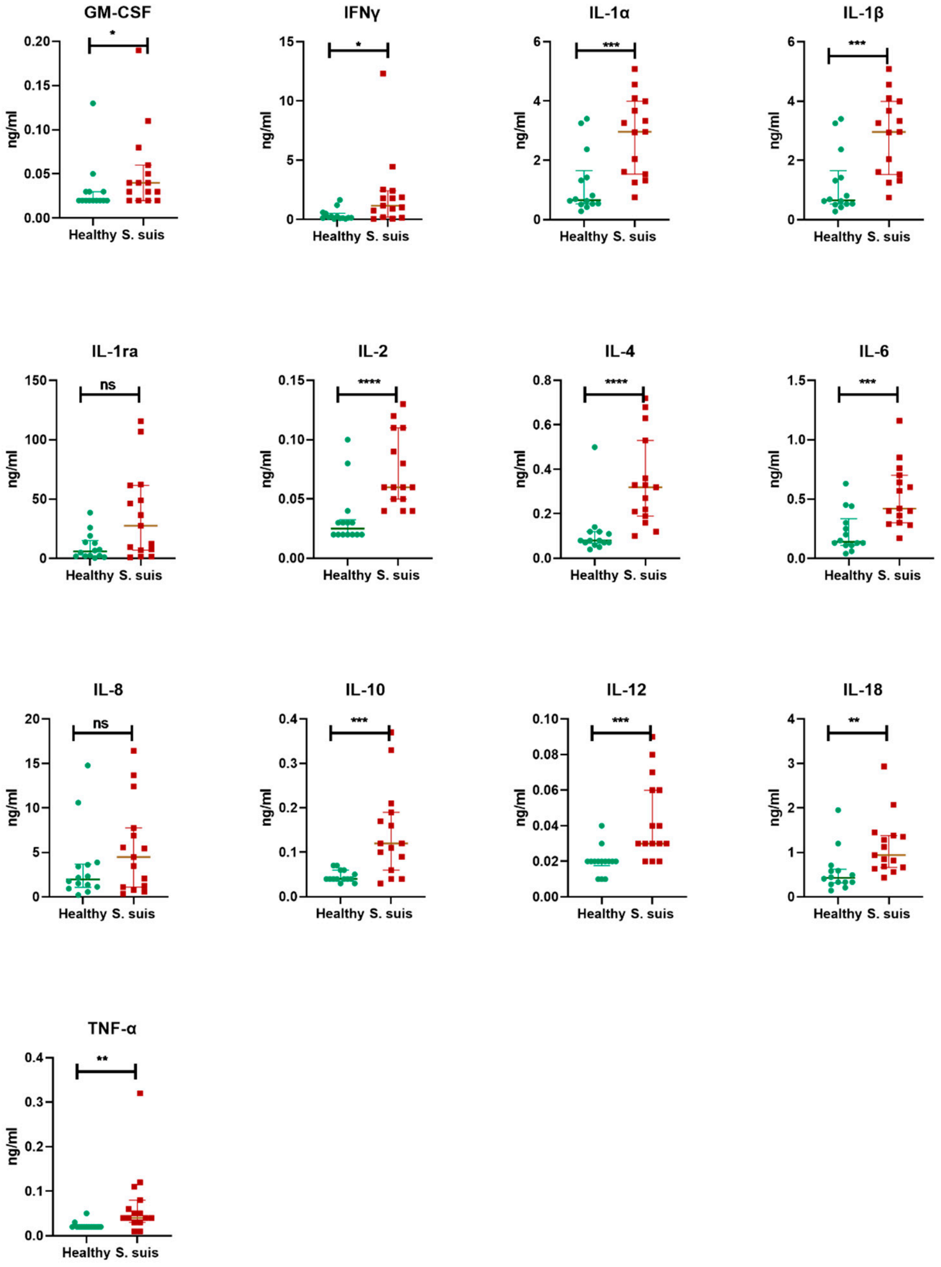


**Figure S1.** Cytokine determinations (ng/mL) in porcine saliva in the group of animals with S. suis infection compared with healthy controls. Individual values are represented as dots and horizontal lines indicated median and 25$^{th}$-75$^{th}$ percentiles values. Cytokines: Granulocyte macrophage colony-stimulating factor (GM-CSF †), interferon-gamma (IFN$\gamma$ †), interleukin (IL)-1$\alpha$, IL-1$\beta$, IL-1ra, IL-2, IL-4, IL-6, IL-8 †, IL-10, IL-12, IL-18, and tumor necrosis factor-alpha (TNF$\alpha$ †). Asterisks highlight statistical significance between the groups (*: $p < 0.05$; **: $p < 0.01$; ***: $p < 0.001$). † GM-CSF, TNF-$\alpha$, IFN$\gamma$ and IL-8 did not yield satisfactory results in the analytical validation.
